# Addressing the elephant in the room: economic growth and the nation’s poor health – changing the economy’s goal for a healthier future

**DOI:** 10.1177/17579139241231883

**Published:** 2024-05-17

**Authors:** RJ Noonan

**Affiliations:** Faculty of Health and Wellbeing, University of Bolton, Deane Road, Bolton BL3 5AB, UK

## Abstract

This opinion piece focuses on how in order to improve the nation’s poor health, the government needs to place more value on social justice and wellbeing as well as the use of regulation to positively change culture and health behaviour.

Life expectancy is stalling,^1^ health inequalities are widening^2^ and crises of obesity,^3^ physical inactivity^4^ and mental health are worsening in the UK.^5^ Yet, there remains a reluctance by the government to discuss the threat of economic growth to health and wellbeing.

The government obsesses about growing the economy. They use the gross domestic product (GDP) – which tallies up the value of goods made and services provided over a period of time – to determine whether ‘they are’ making progress in this respect. But as Robert Kennedy^6^ emphasised in his election speech over 50 years ago, ‘it [GDP] measures everything in short, except that which makes life worthwhile’. It tells us nothing about the health of society.

For instance, what if GDP goes up because more people are consuming more ultra-processed foods more often, resulting in higher rates of obesity? What if GDP goes up because more people are purchasing larger cars and driving them more often instead of walking or cycling, resulting in higher rates of physical inactivity, air pollution and road-related deaths? What if GDP goes up because more people become alcohol dependent or take prescription drugs to deal with the psychological distress brought on by their precarious life? My point is: the economy may grow but what good is this if society becomes sicker?

Much of the preventive effort to tackle public health crises like obesity and mental health has been through the use of educational campaigns like the ‘five a day’ or the ‘five steps to wellbeing’. These well-intentioned health messages compete against corporate advertising promoting junk foods^7^ and materialistic values^8^ – which nudge the publics’ decision making in the opposite direction. When people are content with who they are and what they have, they do not feel the need to consume conspicuously. Advertising drives consumer demand by making people feel that they are missing something. Adverts distort our values and challenge our psychological needs. They promote status competition which drives social anxieties and nonessential material consumption.

To grow the economy each year requires goods to be manufactured faster and services to be delivered faster. Goods and services have to be consumed more often to meet supply and workers have to work faster or for longer. Not just to boost productivity, but to earn the money to meet their rising consumption patterns. Essentially, people need to live their lives faster. No matter what the cost to their health or the environment. One of the easiest ways to feel less stressed is to slow down but slowing down is not an option when the ultimate goal is to grow the economy. Slowing down is a cancer to capitalism.

**Figure:**
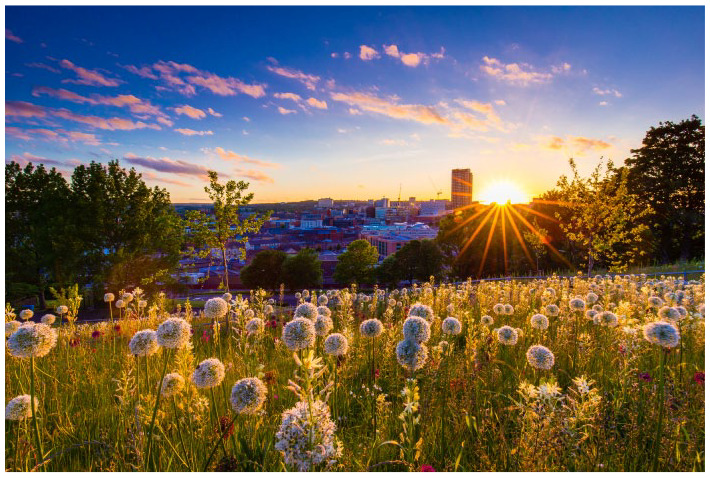


Capitalism is deliberately destructive. It has to be to create the new tastes and new desires to boost consumption, spur on higher rates of production and achieve growth. But to achieve this requires constant innovation and new technology. The social costs of the drive for higher production can be seen in the workplace, with many workers either overworked, losing their jobs to more productive technology, or underemployed – engaged in precarious work.^9,10^ ‘Creative destruction’, which is fundamental to capitalism, not only challenges people’s psychological need for security,^11^ it results in inequality and real hardship for millions.^12^

What is more, capitalism’s thirst for innovation takes the focus away from where it ought to be – solving long-standing societal challenges. In his 1943 seminal paper: *A Theory of Human Motivation*, Abraham Maslow^11^ highlighted how human survival is dependent on satisfying basic needs: clean air and water, nutritious food and adequate shelter. Sure, progress has been made since the work of Maslow but the fact remains: every year millions of people die prematurely from exposure to dirty air and dirty water. Even in the UK – one of the richest nations on the planet – millions lack sufficient nutritious food and rely on food charity.^13^ Millions do not have a stable, decent home, and thousands sleep rough.^14^

The government fixate on sticking plaster approaches to rectify the social and health costs of their growing economy. These treatment approaches are favoured because they align with short-term electoral cycles, vested commercial interests and capitalism’s profit motive. Yet, the solution to poor health is to prevent it from happening in the first place. A long-term vision is needed. Poor health cannot be solved by medicine alone. It is social medicine that people need – proper conditions of life and proper food. Achieving health for all as outlined in the United Nations’^15^ Sustainable Development Goals will take more than baking a bigger cake (i.e. world economy). It will depend on the slices of cake (i.e. wealth and resources) being shared more equally.

Two policy actions could have a big impact on improving population health and wellbeing. The first involves reprogramming the economy by changing its goal.^16^ Social justice and wellbeing have to be valued more than money. It is time to measure health and wellbeing indicators alongside economic targets.

The second involves regulation. Regulation is our country’s most powerful mechanism to tame the impacts of capitalism and positively change culture and health behaviour at scale. Restricting corporate advertisements that challenge health and wellbeing is one option. Another option is to design our streets not for cars but people. Streets are for everyone. They are public spaces. The government needs to ensure that everyone has the opportunity to be active, play, connect and feel safe in their community.

The point of uneconomic growth has been reached – where the social costs of growth are outweighing production benefits. It is time for the Government to consider: what is the purpose of economic growth if millions of citizens still face destitution and do not achieve healthy lives?^2^
